# *Salvadora persica* L.: Toothbrush tree with health benefits and industrial applications – An updated evidence-based review

**DOI:** 10.1016/j.jsps.2021.05.007

**Published:** 2021-06-06

**Authors:** Mohamed Farag, Wael M. Abdel-Mageed, Ali A. El Gamal, Omar A. Basudan

**Affiliations:** a Pharmacognosy Department, College of Pharmacy, King Saud University, Riyadh, Saudi Arabia; b Pharmacognosy Department, Faculty of Pharmacy, Assiut University, Assiut, Egypt; c Pharmacognosy Department, Faculty of Pharmacy, Mansoura University, Mansoura, Egypt

**Keywords:** *Salvadora persica*, Secondary metabolites, Biological potentials, Phytotherapy, Oral care, Validation assays

## Abstract

*Salvadora persica* L. is also known as Arak (in Arabic) and Peelu (in Urdu). Its frequent use as a toothbrush (miswak) is highly recommended by Prophet Muhammad. With a long history in folk medicine for centuries, *S. persica* was used in oral hygiene, food, cosmetics, fuel, and even as a medicine. Previous phytochemical investigation of its different parts afforded different classes of secondary metabolites such as flavonoids, glycosides, sterols, terpenes, carbohydrates and alkaloids. Organic sulfur-containing compounds and elemental sulfur are also present. In addition, there is a huge research on its biological potentials and industrial applications. Many pharmacological activities were reported experimentally, including antimicrobial, antioxidant, analgesic, anthelmintic, anti-inflammatory, antiulcer, sedative, anticonvulsant, anti-osteoporosis, antidiabetic, hypo-lipidemic, in addition to wound-healing, antidepressant and antitumor activities. Recently, a possible activity against COVID-19 protease was documented by molecular docking. This review tries to provide a recent detailed documentation of folk and modern uses of *S. persica,* focusing on the possible relations between its chemical constituents, pharmacological properties, and industrial applications. Moreover, a brief about recent analytical and validation methods for the major antimicrobial component is reported.

## 1 Introduction

Through the ages, nature has served as a valued source of medicinal agents. Currently a lot of natural products are used medically worldwide. Nature can help with new chemical entities of wide structural diversity as templates for semisynthetic and synthetic analogs. The frequent use of natural products or their analogs in the treatment of various diseases can easily be noticed.

Recently, a growing interest in the natural products use, especially those from plants has been clear. Several reasons are behind this interest in plant-derived drugs, including a vast number of chemically and biologically unscreened plants and a long history of traditional medicine, suggesting safety and effectiveness of natural products use. The plant kingdom possesses rich and unique resources for the development of new leads and drugs for widely different pharmacological targets.

*Salvadora persica* is frequently used folk plant. It is widely used by populations worldwide, especially by Muslims. This has attracted our attention to carrying out phytochemical and biological studies on extracts and isolated compounds from *S. persica* as part of efforts to explore therapeutic agents from local plants.

The family *Salvadoraceae* comprises 10 species in three genera [*Azima, Dobera, and Salvadora*], which grow mainly in subtropical and tropical regions of Africa and Asia (Mabberley, 2008). Recently, *Salvadora alii* has been discovered as a new species from Sindh, Pakistan (Tahir et al., 2010). *S. persica* L. [Salvadoraceae] is commonly known under several names across the world, such as Arak, miswak, siwak [Arabic], merge, pilau [India], Caday [Somalia], omungambu [Southern Africa] and toothbrush tree in English (Quattrocchi, 2016).

The name *Salvadora* was first proposed in 1749 by Dr. Laurent Garcin after Juan Salvador Bosca from Barcelona. The name *persica* means Persia (Ahmad and Rajagopal, 2013). The history of miswak use had begun before the Islamic culture. Similar to the cypress tree in English culture, these ancient trees were frequently associated with graveyards in Pakistan.

With a long history in folk medicine for centuries, *S. persica* was used in oral hygiene, food, cosmetics, fuel, and even as a medicine. Thus, leaves and fruits were consumed as green vegetables or in salads. The resin may be used for varnish manufacture. Also leaves and young twigs were useful as a nutrient for several animals, including cows, camels, goats, and sheep to enhance cow lactation and increase the general body weight of animals. It has been reported that honey from *S. persica* has medicinal value, and its flowers are a good source of the honey bee nectar (Orwa et al., 2009, Sher and Alyemeni, 2011).

Moreover, *S. persica* has an amazing history of use as a medicine by many ethnic groups, particularly in Africa and Asia. All parts of the plant, including flowers, fruits, leaves, bark, seeds, stems, and roots, have been used to treat many disorders related to various physiological systems in human beings including, circulatory, motor, excretory, digestive systems (Aumeeruddy et al., 2017).

*S. persica* was also used by ancient Arabs for teeth whitening and to make them healthy. Prophet Muhammad around 543 CE recommended this custom (Dutta and Shaikh, 2012). Moreover, the Prophet used miswak very often; whenever he woke up from sleep, before prayers, when he would enter his home, showing how important it is to use it frequently for good oral hygiene.

Botanically, *S. persica* is a perennial evergreen halophyte. It can grow under extreme conditions, from very dry environments to highly saline soils (Maggio et al., 2000). It is considered a small tree or a shrub as its height is up to 10 m, with a diameter up to three feet. The main stem is more than one foot in diameter, erect or trailing with profusely drooping and straggling branches. Young branches are green. The bark is slightly rough and greyish brown on the main stem, paler elsewhere (Panday, 2004). The habitats are in littoral forests and saline soils, and the plant is commonly found in Sindh [Pakistan], dried parts of Ceylon and India, as well as in dry regions of Egypt, West Asia, and Abyssinia (Khan and Qaiser, 2006).

## 2 Phytochemical profile

All parts [root, seed, flower, fruit, leaf, bark, stem, and twigs] of *S. persica* have been screened. Extensive phytochemical analysis revealed the presence of carbohydrates, flavonoids, terpenes, sterols, alkaloids and glycosides and. Organic sulfur compounds and elemental sulfur are also present as well as small amounts of fluoride, calcium, phosphorus, silica, and ascorbic acid (Aumeeruddy et al., 2017).

### 2.1 Volatile components and sterols

**Fig. 1:**
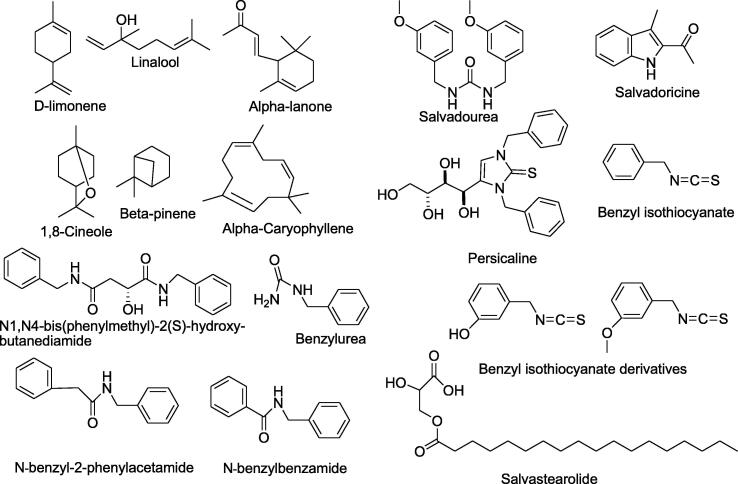
Some important chemical constituents of *S. persica.*

The percentage of the volatile oil is 0.16% with heptadecane β-carbonic acid as a major component (Daxenbichler et al., 1991). More volatile components, as shown in Fig. 1, are hydreate linalool, D-limonene, linalool acetate, phenylmethanal, decenaldehyde, methyl nonyl ketone, 7-hydroxy-delta(1)-tetrahydrocannabinol, 2-Cyclohexenone, 3-methyl-6-(1-methylethenyl), 1-(2,6,6-Trimethylcyclohexa-1,3-dien-1-yl)but-2-en-1-one, iraldeine, indole, hedycaryol, 2-pentadecanone-6,10,14-trimethyl, pelargonic acid, delta-silinene, eudesm-4(14)-en-11-ol, tricosane, octadecanoic acid, dedecanoic acid, nonadecane, tetra dedecanoic acid, 2-hexadecen-1-ol-3,7,11,15-tetramethyl, 9-octadecanoic acid, 9,12-octadedecenoic acid, and hexadecanoic acid (AbdELRahman et al., 2003).

Roots, stems, and seeds of *S. persica* have been reported to contain volatile compounds such as *cis*-2-methylcyclohexanol, benzaldehyde, chloromethylbenzene, benzylalcohol, 3-methyl-2-furancarboxylic acid, *N*-acetylpiperidine, 3,5-dihydroxy-6-methyl-2,3-dihydropyran-4-one, amyl benzoate, nonanal, pyridine derivatives, tetradec-1-ene, stearyl alcohol, 3,4,5-trimethoxyphenol, tetradecanoic acid, elaidic acid, hexadecanoic acid, (*Z*)-9-octadecenoic acid, and (*Z*)-9-octadecenoic acid ethyl ester. Essential oils from leaves contain eugenol, isothymol, thymol, benzyl Nitrile, β-caryophyllene, isoterpinolene, and eucalyptol as the major components (Alali and Al-Lafi, 2003). The hydrodistillation of the stem gives a mixture of oxygenated monoterpenes (54%), sesquiterpene hydrocarbons (21%), and monoterpene hydrocarbons (11%). Among these, eucalyptol (46%), humulene (13.4%), Pseudopinene (6.3%), and 9-*epi*-(*E*)-caryophyllene were identified as the major compounds (Alali et al., 2005).

Many recent studies have reported new compounds found in different parts of this plant. For instance, new steroid esters, β-sitosteryl-3-vanilloyl-4′-palmitate, β-sitosteryl arabinosyl vanilloyl stearate, β-sitosteryl vanilloyl oleate, and β-sitosteryl-3-vanilloyl-4′-stearate, have been recently obtained from the roots (Khan et al., 2018).

### 2.2 Alkaloids and nitrogenous compounds

Roots are rich in salvadourea, and benzyl isothiocyanate (Fig. 1) which shows antiviral activity against a dangerous oral cavity virus, herpes simplex virus (Kamil et al., 1999). In addition, a high content of alkaloids such as salvadoricine, as well as trimethylamine (Fig. 1) were found in the roots (lafi Al and Ababneh, 1995). Also, pyrrolidine, pyrrole, and derivatives of piperidine have been isolated from the sticks of *S. persica* which are nitrogen-containing compounds (Galletti et al., 1993).

The four benzylamides shown in Fig. 1, N-benzylbenzamide, N-benzyl-2- phenylacetamide, benzylurea, and butanediamide-N,N-bis-phenylmethyl-2-hydroxy-butanediamide, were purified from the plant stem (Khalil, 2006).

Extracts of twigs, stems and roots have been reported to contain high amounts of salvadourea (Khalil, 2006). Some alkaloids such as theobromine, caffeine, and trigonelline were also found in the bark (Farag et al., 2017).

Interestingly, this majestic plant still yields new bioactive compounds until now. Our team (Farag et al., 2018) recently has isolated a new antioxidant sulfur-containing imidazoline alkaloid, persicaline, from the roots.

In another recent study from Saudi Arabia (Abdel-Kader et al., 2017a, Abdel-Kader et al., 2017b, Abd El Kader et al., 2017c), the major antimicrobial compound benzyl isothiocyanate and two new antimicrobial derivatives, 3-hydroxy benzyl isothiocyanate and 3-methoxy benzyl isothiocyanate (Fig. 1) were isolated from the roots of *S. persica*.

Furthermore, the phytochemical compositions of three *S. persica* root samples and one stem sample of various regions (Saudi Arabia and Egypt) were compared by (Farag et al., 2017). More amino acids (2–12%) were detected in the roots, while only 1% was found in the stem. L-alanine is considered the major amino acid with a percentage of (1–10%), while other amino acids were present at comparable levels. Total nitrogenous compounds were also found at higher levels in the roots, ranging from 3.2 to 5%, as compared to 2.86% in the stem. Among these, urea was mostly present in the stem, while *N*-benzylamine in the roots. Differences in the composition, due to geographical locations, were confirmed by differences in the phytochemical ingredients included in the three root samples (one from Egypt and two from Saudi Arabia).

It is important to notice that many factors can contribute to differences in phytochemical constituents of this plant, such as climatic conditions and geographical origin, as well as different agricultural and extraction techniques applied. Supporting this idea was a study by Al-Ghamdi and El-Zohri (Al-Ghamdi and El-Zohri, 2017), which documented variations in phytochemical constituents of *S. persica* roots and leaves from two various regions [Shada Mountain and Al-ahsabah valley]. The quality of soil in the Al-ahsabah valley, located in the southwest of Saudi Arabia, differed from that of the Shada Mountain in containing lower amounts of fine and coarse sand and higher amounts of clay and silt, in addition to exhibiting higher moisture, pH, and organic matter than those in the Shada Mountain soil.

Moreover, the ratios of root colonization and mycorrhizal spores were lower in Al-ahsabah. Amino acid, protein, and carbohydrate amounts in the leaves and roots of *S. persica* harvested from the Shada Mountain were significantly lower than those in the leaves and roots harvested from the Al-ahsabah valley. These findings show the importance of studying all factors affecting *S. persica* chemical constitution and its bioactivity to get more potent and better quality extracts.

### 2.3 Glycosides and phenolic compounds

The roots of both Egyptian and Saudi plants have yielded two glucosinolates; glucotropaelin and sinigrin (Abdel-Wahab et al., 1990, Ezmirly and El-Nasr, 1981). Salvadoside, salvadoraside, syringin, liriodendrin, and lignan glycosides were also isolated from the stem (Ohtani et al., 1992). Cyanogenic glycosides are also present (Khalil, 2006).

Another study has reported the main phenolic compounds in the root as 4,5-*O*-D-caffeoylquinic acid and 5-*O*-caffeoylquinic acid while 3,5-*O*-D-caffeoylquinic acid, 5-*O*-caffeoylquinic acid, epicatechin, and catechin were high in the stem. Roots are also rich in *m*-anisic acid (Kamil et al., 1999). A high percentage of naringenine, 5-O-caffeoylquinic acid, was also found in the bark (Farag et al., 2017).

### 2.4 Fixed oil and vitamins

Seeds of *S. persica* contain approximately 40% of oil, composed of myristic (55%), lauric (20%), cetylic acid (20%), and *cis*-9-Octadecenoic acid (5%) fatty acids, which are excellent-soap components (Ahmed et al., 2008). In addition, (El-Desouky et al., 2018) have isolated salvastearolide, a new stearic acid ester in Fig. 1, from the *n*-hexane fraction of the seeds. The seed coat and leaves have been noticed to contain fatty acid methyl esters, tocopherols (α-tocopherol, γ-tocopherol, vitamin E and γ-tocotrienols), sterols (β-sitosterol, phytosterol, stigmasterol, campestrol, and 5-avenastrol), and phenolic compounds.

Moreover, roots are rich in elemental sulfur (Kamil et al., 1999). Extracts of twigs, stems and roots have been reported to NaCl and KCl, flavonoids such as rutin, tannins and saponin (Khalil, 2006).

It is also important to state that not only the plant tissues of *S. persica* are responsible for the phytochemicals isolated from this plant but also some endophytic fungi were found to produce their secondary metabolites in the medium. Although few studies have been done to isolate endophytic fungi of *S. persica*, (Korejo et al., 2014) have discovered eight endophytic fungi (including two *Aspergillus* spp., three *Penicillium* spp., *Fusarium solani*, *M. phaseolina* and *Rhizoctonia solani*) from seventy four stem, root and leaves of *Salvadora* species. Recently, forty-two fungal isolates were collected from 135 young and old stem and 125 root segments by (Elgorban et al., 2019). Those 42 isolates representing ten fungi include: *Trichoderma* sp. [the most common], two species of *Alternaria*, *R. arrhizus* and *Aspergillus* sp. and six sterile mycelia. They were grown in liquid culture and their crude extracts were examined against pathogenic fungi and bacteria. Thirty-seven active compounds were isolated from the crude extracts of *Alternaria* sp. [A8] and identified using GC–MS. Thirteen major bioactive compounds were reported and revealed strong antibacterial activity in combination. By phylogenetic analyses, the fungal isolate was identified based on LSU rDNA sequence data and it might be undescribed species of *Alternaria* (Elgorban et al., 2019).

Based on the above data, each part of *S. persica* may be expected to contain pharmaceutically important phytochemicals that can contribute to the health of humans and animals. The presence of micro- and macronutrients, along with the above-mentioned phytochemicals, helps correlate the nutritive, traditional, and biological properties reported. A future investigation may result in the discovery of new bioactive compounds in *S. persica*, leading to the formulation of more potent drugs and/or new bioproducts.

## 3 Health benefits of the plant and its extracts

Various biological activities of *S. persica* have been recorded using different extracts and their fractions from each part of the plant, as well as oil. Traditionally, crude extracts of several parts of *S. persica* have been utilized for centuries as a drug for many diseases, as mentioned before. Great efforts have been made to investigate these activities and to understand the mechanisms of action, based on the chemical constituents. Many pharmacological activities were evaluated experimentally, including antimicrobial, anthelmintic, analgesic, anti-inflammatory, antiulcer, antioxidant, anticonvulsant, sedative, anti-osteoporosis, antidiabetic, hypolipidemic, in addition to wound-healing, antidepressant and antitumor activities. This section provides an insight, with critical analysis, into the plant’s pharmacological activities in relation to its traditional uses and phytochemistry.

### 3.1 Antimicrobial activity

Much effort has been made to confirm the antimicrobial activity of *S. persica* versus a broad range of microorganisms (Aumeeruddy et al., 2017). Research has indicated that compounds and minerals with antibacterial activities against various species of the oral cavity cariogenic bacteria are present in *S. persica.* These constituents showed ability to inhibit cariogenic bacteria growth and acid production. In addition, substances from this plant possess plaque-inhibiting properties. Miswak parts were tested either in suspension, applied to agar plates, or embedded in agar. Stronger or comparable effects were noticed for suspended miswak compared with those of miswak embedded in agar, although in both cases, the plant showed strong antibacterial activities against all tested bacteria.

Recently, the fruit extract has shown selective antimicrobial activity for *Streptococcus* mutants isolates with minimum inhibitory concentration [MIC] and minimum bactericidal concentration [MBC] values of 3.12 and 6.25 mg/mL, respectively (Al Bratty et al., 2020). The effect of essential oils from dried and fresh roots of *S. persica* have been studied by (Khan et al., 2020). The oils showed IC_50_ values comparable to that of chlorhexidine digluconate.

Also, (Khalil et al., 2019) documented that methanol extract of *S. persica* had significant antibacterial effect versus *S. aureus* and *Streptococcus* species isolates, and it may be gave a good alternative method for controlling oral pathogen. Interestingly, four compounds; apigenin, luteolin, astragalin and kaempferol-3-*O*-rhamnoside have been isolated from *s. persica* showed good anti-MRSA [Methicillin-resistant Staphylococcus aureus]. This type of bacteria is resistant to many antibiotics and can cause dangerous diseases like pneumonia and sepsis. The isolated compounds gave reasonable anti-MRSA activities with IC_50_ values of 10.3, 11.5, 3.5, and 4.5 μg/mL, respectively (Ghoneim et al., 2019). However, the aqueous extract was found to be less active than the alcoholic extract of *S. persica* (Al-Sabawi et al., 2007).

The antimicrobial action of *S. persica* can be due to its wide variety of phytochemical components. One of them is benzyl isothiocyanate, the major compound from the roots, which showed a significant effect versus gram-negative bacteria. It has lipophilic and electrophilic properties, which may confer it the ability to penetrate the outer membrane of bacteria and may suppress the bacterial redox system by damaging the membrane potential (Sofrata et al., 2011). A significantly higher antibacterial activity also has been resulted from mixture of *S. persica* with antibiotics than that of each individual treatment (Ahmed et al., 2010).

### 3.2 Oral health

Many studies have documented a significant action of miswak as an anti-gingivitis, anti-plaque, anti-cariogenic, promotion of gingival wound healing, whitening properties, orthodontic chain preservation, and biocompatibility with oral cells. Different forms of miswak helped in oral health maintenance and management (Nordin et al., 2020).

#### 3.2.1 Clinical studies

A recent report by (Landu et al., 2020) showed significant use of *S. persica* stick in decreasing the number of oral pathogen colons in mechanically ventilated patients (p < 0.05) which means that *S. persica* oral care can help patients with mechanical ventilation. Also a new clinical trial by (Sabbagh et al., 2020) reported ability of *S. persica* to alter the nature of salivary bacteria in favor of less risk species in school children group of study.

Another recent clinical study by (Rifaey et al., 2020) has studied miswak sticks efficacy as an oral hygiene aid. They randomly assigned twenty healthy volunteers into two equal groups. Group 1 were instructed to use both toothbrush and miswak [TB + M] for the first 2 weeks from baseline [T1] and only toothbrush for the next 2 weeks [T2]; and Group 2, only TB during T1 and TB + M during T2. They have evaluated gingival index (GI), plaque index (PI) and bleeding on probing (BOP) were at baseline [T0], T1 and T2 visits. They concluded from their single center, single-examiner blind, randomized, cross-over study that oral hygiene and gingival health may be improved by complementing miswak chewing sticks with tooth brushing.

This finding was supported by another older study by (Patel et al., 2012) in which 30 systemically healthy subjects diagnosed with mild to moderate gingivitis were divided into three equal groups; Group A [toothbrush users], group B [toothbrush and miswak users], and group C [miswak users]. Subjects were advised to use toothbrush, miswak, or both, three times daily with gingival index, Plaque index recorded in addition to taking digital photographs of the total labial surfaces of the teeth for image analysis. Recording of data were done at baseline, 2nd, 4th, 6th, and 8th week time intervals. The randomized, single-blind, parallel-armed study concluded that significant improvement in plaque score and gingival health were recorded when miswak was used as an adjunct to tooth brushing.

Moreover, effect of chewing *persica* [miswak] as antibacterial was proved by many studies. For example, chewing *persica* containing gum liberated fluoride ions in saliva and increased saliva flow rate comparable with NaF chewing gum use as a gold standard (Mortazavi et al., 2019). Also, addition of chlorhexidine and miswak extract to conventional glass ionomer has good impact on its clinical performance and antibacterial properties as proved by an *in vivo* study by (Kabil et al., 2017).

It is also important to mention here that a recent *meta*-analysis study by (Jassoma et al., 2019) concluded that *S. persica* extract use caused a significant reduction in the plaque score and cariogenic bacterial count. This reduction was less than that recorded by chlorhexidine mouthwash but due to *S. persica*-containing rinse’s considerable efficacy, safety and cost-effectiveness, it could be a suitable oral hygiene alternative for a long-term use.

A study by (Abdulbaqi et al., 2016) has demonstrated that the mixture of the *S. persica* root stick and green tea (leaves of *Camellia sinensis* L.) exhibited synergistic effects versus primary colonizers of dental plaque and *in vitro* antiplaque activity. This research showed the importance of testing the efficacy of combinations of *S. persica* with other medicinal plants, mainly whose traditional use is reported for oral care. This may potentially produce new effective polyherbal formulations.

Another study by (Azaripour et al., 2017) has evaluated the efficacy of a miswak extract containing toothpaste on gingival inflammation in comparison with that of a herbal and a conventional toothpaste. They found that The miswak extract-containing toothpaste showed a similar effect as the herbal toothpaste and can be safely used for domestic oral hygiene in patients with gingivitis.

The toothbrush has been reported to be less effective than miswak in reducing gingivitis in a cross-sectional observational descriptive research done on 528 subjects (63.6% females and 36.4% males) who used a toothbrush alone or combined with a toothpaste (Shetty et al., 2010).

Another research was done in Sudan and included male toothbrush users (n = 104) and miswak users (n = 109) in the age range of 20–65 years (Darout et al., 2000). They have reported that significantly less dental calculus, fewer sites with a probing depth of ≥ 4 mm, and less clinical attachment loss ≥ 4 mm, as well as a tendency for less gingival bleeding in the posterior sextants, were recorded in the miswak users compared to the toothbrush users. The results confirmed that the periodontal status of the miswak users in the Sudanese population was better than that of the toothbrush users, although non-significant variances were observed in the anterior sextants.

In addition, (Al-otaibi et al., 2003) have found in a crossover single-blinded randomized clinical study in fifteen healthy Saudi Arabian male volunteers, aged 21 to 36 years old, who properly used miswak or a toothbrush, that the toothbrush was less effective than miswak in reducing plaque formation and gingivitis.

#### 3.2.2 Animal experiment

Recently a study by (Faruk et al., 2020) evaluated the healing effects of topical *S. persica* (SP) extract, low-level laser (LLL) and high-level laser (HLL) therapies in a rabbit model of caustic-induced tongue ulcers. They found that topical SP extract, LLL and HLL were equally effective therapies against caustics-induced tongue ulcers. However, they recommended SP extract because of its safety, availability and low cost.

### 3.3 Wound-healing activity

Wound-healing is a complicated multifactorial process that ends in the contraction and closure of the wound and a functional barrier recovery. *S. persica* contain phytoconstituents like tannins, saponins, flavonoids and sterols which were found to assist wound healing because of their antioxidant and antimicrobial activities. This was proved by (Tatke et al., 2017) as they formulated the methanol extract into a gel and tested its wound healing activity in rats. It was found that gel containing methanol extract of miswak twig possess significant wound healing activity.

### 3.4 Antioxidant activity

Excessive free radicals in the body can lead to oxidative stress and may cause many chronic diseases (Pham-Huy et al., 2008). Fortunately, the *S. persica* antioxidant potency has been demonstrated via an impressive collection of standard tests. A recent evidence has been obtained by (Farag et al., 2018) who documented antioxidant activities of an ethanol extract, its many fractions, and some isolated compounds, in particular of a new compound, persicaline. Various *in vitro* assays, such as 1,1-diphenyl-2-picrylhydrazyl [DPPH], superoxide anion, and nitric oxide radical-scavenging methods, have been utilized to estimate the antioxidant activities of the fractions and isolated compounds. Persicaline exhibited a promising antioxidant activity, with half-maximal inhibitory concentrations [IC_50_] of 0.1, 0.08, and 0.09 µM in the three assays, respectively, which were comparable to those of vitamin C.

Another study by (Mekonnen, 2018) found that *S. persica* root and stem extracts have good amount of total phenols and small amount of flavonoids which have the major role in oxidation control. (Qasim et al., 2016) research has demonstrated that acetone and chloroform were less effective than other solvents, such as methanol, ethanol, and water, for extracting *S. persica* antioxidant compounds. Chloroform was the least effective, while methanol was the most effective. Also, methanol extract showed the strongest DPPH-scavenging activities (IC_50_ = 20.92 μg/mL).

The highest linkage between the polyphenol content and antioxidant activity [measured by the DPPH assay] was found for the methanol extract, followed by the ethanol and water extracts, while a weaker relation was noticed for the chloroform and acetone extracts. These findings confirm a higher extraction affinity of polar solvents over non-polar solvents for active antioxidant ingredients from *S. persica*.

Moreover, a higher DPPH free radical-scavenging activity and a higher ferric ion (Fe^3+^)-reducing antioxidant power [FRAP] were shown by an ethyl acetate extract of leaves [IC_50_ = 11.8 and 480 μg/mL, respectively] compared with those shown by a stem extract [IC_50_ = 7,817 and 20,124 μg/mL, respectively] from *S. persica* trees growing in Ahaggar in southern Algeria (Kholkhal et al., 2010).

The higher total phenolic, tannin, and flavonoid contents found in leaves compared with those found in the stem can indicate a higher activity of leaves. However, a butanol extract of the stem revealed a greater antioxidant activity [DPPH: IC_50_ = 14 μg/mL; FRAP: IC_50_ = 3,290 μg/mL] than that shown by a leaf extract [DPPH: IC_50_ = 257 μg/mL; FRAP: IC_50_ = 3,660 μg/mL].

The reasons for this difference in results may include an easy extraction of antioxidant enzymes with butanol and the quality and quantity of phenolic compounds in different extracts (Kholkhal et al., 2010).

Additionally, *S. persica s*tem extracts were less effective than root extracts in scavenging DPPH, reducing the ferric ion, and inhibiting lipid peroxidation in a rat brain homogenate. Meanwhile, hydro-alcoholic extracts were more effective antioxidant extracts than aqueous and alcoholic extracts (HOODA and SINGH, 2012). Therefore, we strongly recommend further studies to estimate the impact of extraction solvents on the quality and quantity of antioxidant compounds.

### 3.5 Anthelmintic activity

Parasites can infect human which is still a big problem worldwide. A lot of medicinal plants helped to treat this problem. A research group of Dkhil have evaluated the anthelmintic activity of three doses; 200, 100 and 50 mg/m L of *S. persica* root extract [SE] (Dkhil et al., 2019). They have used albendazole as a reference drug and *Allolobophora caliginosa* as a model worm.

To evaluate the extract’s anticoccideal activity, they infected a group of mice with *Eimeria papillata* sporulated oocysts. Mice were treated with SE at dose of 300 mg/Kg for 5 days. The extract decreased the number of meronts and gamonts of the parasite in jejunum. Also, it modulated glutathione and malondialdehyde levels and the catalase activity as well. They concluded that *S. persica* exerted a powerful antioxidant, anthelmintic and anti-coccidial activity.

In addition, (El-Bali et al., 2020) have recently evaluated the antiprotozoal potential of *S. persica in vitro* versus *Blastocystis* sp. human isolates which is a group of anaerobic protozoa live in the gastrointestinal tract of humans and a broad variety of animals. *Blastocystis* sp. positive stool samples were taken from patients with gastrointestinal complaints and asymptomatic individuals diagnosed by microscopy were furthermore cultured *in vitro* and characterized by PCR and multiplex-PCR using sequence-tagged-site primers to detect their subtypes. Antiprotozoal activity of untreated and heat-treated *S. persica* roots aqueous extracts was examined *in vitro* by serial dilutions on three *Blastocystis* sp. subtypes; ST1, ST3, and ST5 isolated from symptomatic patients.

A significant antiprotozoal activity was recorded with both, untreated and heat-treated aqueous extracts of *S. persica* at minimal concentration of 2.5 µl/ml compared to parasites’ growth controls (P < 0.05). Maximal effect was noticed at a concentration of 20 µl/ml of *S. persica* aqueous extract. Therefore, they concluded that heat-stable components in aqueous roots extract of *S. persica* have significant antiprotozoal activity against *Blastocystis* sp. subtypes ST1, ST3, and ST5 *in vitro*. Further efforts are requested to isolate and elucidate the active antiprotozoal compounds of *S. persica* roots and evaluate them *in vivo*.

### 3.6 Anti-inflammatory activity

A clear anti-inflammatory action against carrageenan-induced paw edema in rats has been reported for an *S. persica* leaf ethyl acetate extract [500 mg/kg, orally], which was near to or slightly less effective than the indomethacin [10 mg/kg, p.o.] as standard drug (Rajesh et al., 2010). Meanwhile, (Ibrahim et al., 2011b) have reported that the thickness of edema in rats was significantly reduced, in a time-dependent manner, after ingestion 100 mg/kg of an *S. persica* stick crude ethanol extract and its ethyl acetate fraction.

Flavonoids present in the ethyl acetate fraction may be main contributors to the *S. persica* anti-inflammatory activity. Rutin, apigenin rhamnoglucoside, and luteolin glucoside were the three major flavonoids identified in this fraction.

From the above studies, we can state that *S. persica* leaves displays a noticed anti-inflammatory activity. More work is required to test other parts of the plant for possible activity. Besides, other *in vitro* and *in vivo* assays should be used to evaluate the safety of *S. persica* as an anti-inflammatory drug. However, the exact mechanism of the documented *S. persica* anti-inflammatory activity still needs to be elucidated in further in-depth studies.

### 3.7 Health benefits for reproductive system

A report by (Rabbani et al., 2019) stated that the decoction *of S. persica* can reduce the somatic and germinal cell nuclear damages induced by cigarette smoke exposure in rats and the significant (P < 0.05) activity was observed at 200 mg/kg dose. Moreover, (Soliman et al., 2017) observed that *S. persica* roots extract attenuated lead acetate-induced testicular oxidative stress in rats significantly at doses of 250 and 500 mg/kg.

### 3.8 Analgesic activity

Evaluation of the analgesic activity of root hydro-alcohol extract of *S. persica* on mice has been done by (Hooda and Pal, 2017) by eddy's hot plate and tail immersion methods. They found that *S. persica* root crude hydro-alc. extract at doses of 100 mg/Kg, 200 mg /Kg and 400 mg /Kg had significant analgesic activity.

Also, it have been documented that in three analgesic tests, the response to thermal and chemical stimuli was reduced in mice intraperitoneally injected with decoction of *S. persica* (Sulaiman et al., 1996). The suppression effect was higher against the thermal stimuli than against the chemical stimuli. The response to chemical stimuli in a writhing reflex test is usually mediated via visceral receptors, while that to thermal stimuli is mediated via skin pain receptors. Therefore, it is predicted that miswak is more active for peripheral pain than visceral pain. These findings may support the traditional use of miswak decoctions by application to the oral mucosa for treating oral pain.

Moreover, a research by (HOOR et al., 2011) have recorded the crude ethanol extract of *S. persica* showed a dose-dependent analgesic activity in mice at tested oral doses of 500 and 700 mg/kg body weight. However, the tested part of *S. persica* was not specified which was the important drawback of this study. The data indicated that the latency period in a tail-flick test was significantly higher (approximately 40–50% on average) in *S. persica*-treated mice than in the control group and was similar to that obtained with aspirin. The analgesic activities of the extract and aspirin was found maximum at 120–150 min after gradual increase then declined afterward.

The mechanism for miswak analgesic action is unclear. However, interaction with opiated system was the assumed mechanism since analgesic action of miswak was antagonized by naloxone.

### 3.9 Antiulcer activity

Gastric ulcers are frequent diseases nowadays. Alcohol consumption is one of the major causes of that disease. A study by (Lebda et al., 2018) examined the protective action of *S. persica* extract against ethanol-induced gastric ulcer. Gross and microscopic examinations of the EtOH-treated group revealed severe gastric hemorrhagic necrosis, submucosal edema, destruction of epithelial cells and reduced glycoprotein content at the mucus surface. These pathological lesions were improved after treatment with *S. persica* extract. They concluded that *S. persica* extract showed significant antiulcer activity in ethanol-induced gastric ulcer rat models via antioxidant activity, mitigation of pro-inflammatory cytokines, and apoptosis, as well as remodeling of both nitric oxide synthase isoforms.

A study by (Sanogo et al., 1999) have reported that a lyophilized decoction from *S. persica* twigs (at a dose 500 mg/kg body weight by gavage) showed an antiulcer activity in male wistar rats. Optical microscopy showed that the elements of the gastric mucosa were restored in the treated rats. Normal distribution of the glands and lamina propria tended to recover, although the epithelial coat was not completely restored.

In addition, an antiulcer activity of a lyophilized decoction of *S. persica* stems was investigated by (Monforte et al., 2001) in an acetylsalicylic acid-induced ulcer male Wistar rat model. The study reported a significant reduction in an ulcer index [0.9] compared with that in the control group [11.4] after treatment with the lyophilized decoction once a day for 7 days. Histologically, the glandular tissue showed considerable regeneration, and some variances were recorded in many organelles of parietal cells after treatment.

The limitations of these studies were that none have tried to link the antiulcer potential of *S. persica* to its phytochemical composition. Therefore, it is strongly recommended that more studies are performed to investigate the antiulcer action of *S. persica* and elucidate its mechanism, as related to the phytochemical composition.

### 3.10 Anticonvulsant, sedative and antidepressant activities

Recently, (Rabbani, 2020) has evaluated the anxiolytic and antidepressant activity of *S. persica* in cigarette smoke-induced neurobehavioral changes in rats. The observation from this study showed that exposure of cigarette smoke for eight weeks significantly enhanced the experimental parameters of depression and anxiety, besides increasing the monoamine oxidase-A and relative brain weight when compared with control animals. Administration of *S. persica* lyophilized decoction exhibited a dose-dependent significant inhibition in the neurobehavioral parameters. The treatment was also found to reduce the serum monoamine oxidase- A and relative brain weight in the cigarette smoked animals. He concluded that decoction of *S. persica* might has antidepressant and anxiolytic properties in the cigarette smoke exposed animals. These actions could be related to its antioxidant and reversal in the neurocircuilatory changes induced by the cigarette smoke.

In addition**,** Stem extracts of *S. persica* were screened for possible anticonvulsant and sedative effects. Potentiating activity of sodium pentobarbital and effects on generalized tonic–clonic seizures, produced by pentylene tetrazole in rats, have been documented.

It has been reported that the sleeping time was extended and the induction time of sleep, induced by sodium pentobarbital, decreased. Pentylene tetrazole-induced convulsions were also prevented by the *S. persica* extracts. The latency period increased, and the death rate was diminished (Monforte et al., 2002). It is important to notice that the chloroform extract was the most active. From this research this plant can be suggested as another source of potential insomnia drugs, besides its ability to reduce the problems of other sedative drugs.

### 3.11 Locomotor activity

Potential effects of *S. persica* extracts on mouse stereotype movements and exploratory locomotor activities have been investigated. A significantly lower exploratory locomotor activity was recorded in the mice injected with the extracts than that in the controls. In addition, a significantly lower number of stereotype movements was displayed by the mice injected with the extracts (Dutta and Shaikh, 2012).

### 3.12 Anti-osteoporosis

*S. persica* stick extract efficacy in an ovary-ectomized rat model of osteoporosis was investigated by (Fouda and Youssef, 2017) . The extract was taken p.o., at doses of 50, 150, and 300 mg/kg, dissolved in of distilled water to ovary-ectomized female Sprague–Dawley rats every morning for 16 weeks. A dose-dependent protective action was displayed by the extract in the range from 50 to 300 mg/kg/day.

This preliminary research is the only study documenting an anti-osteoporosis activity of the *S. persica* stick, although the activity was not traditionally reported in an indigenous population. Deeper studies are highly recommended to discover the potential antiosteoporosis effect of other parts of *S. persica*, and more trials should be conducted to isolate bioactive compounds responsible for this effect.

### 3.13 Hypolipidemic and hypoglycemic activities

Hypoglycemic and hypolipidemic effects of *S. persica* have been investigated by several researchers and were also assessed in a diet-induced rat hypercholesterolemia model. Triglycerides, [HDL] cholesterol, [LDL] cholesterol, and plasma levels of total cholesterol [TC] were measured. Significant decreases in the plasma levels of TC and LDL cholesterol were found in rats after treatment with an *S. persica* decoction, and the effects were more obvious after 30 days of treatment (Galati et al., 1999).

A report by (Khan et al., 2014) has documented variations in TG and HDL concentrations in male albino Wistar rats. Among many extracts of *S. persica* root tested (Indian vs. Arabian *S. persica*, at doses of 250 and 500 mg/kg, the aqueous root extract of Arabian *S. persica*, when administered as an aqueous suspension at a dose of 500 mg/kg once daily for 14 days, after seven days of streptozotocin treatment [60 mg/kg], caused a better decrease [77%] in blood glucose than did a standard drug, glibenclamide, which was tested at 5 mg/kg/day, p.o. At the end of week 4, biochemical tests performed in the Arabian *S. persica* extract 500 mg/kg-treated rats also demonstrated significant reductions in TGs, TC, LDL and very LDL [VLDL] cholesterol and an increase in HDL cholesterol, compared with the respective levels in the diabetic control rats.

Moreover, the TG level in the Arabian *S. persica* extract-treated rats [75.40 mg/dL] was better than that in the normal [non-diabetic] control rats [87.34 mg/dL], while the VLDL cholesterol levels were comparable between the two groups [22.78 and 21.55 mg/dL, respectively]. Notably, this extract was comparable to or slightly less effective than the standard drug glibenclamide, tested at 5 mg/kg/day [p.o.], in the extent of reductions in TG, TC, LDL and VLDL cholesterol and an increase in the HDL cholesterol level, compared with those in the diabetic control. The blood glucose level was also decreased in diabetic adult Wistar albino rats by a hydroalcoholic (70% ethanol) roots extract of *S. persica*, as reported by (Hooda et al., 2014).

A new study by (El Rabey et al., 2017) evaluated antioxidant, antidiabetic and hypolipidemic activities of *S. persica* twigs powder in the diabetic male rat. They divided twenty-four male albino rats into four groups; the first group was untreated control, the second was the diabetic positive control, and the third and the fourth were diabetic treated with 10 g/kg and 20 g/kg *S. persica* twig powder in the diet, respectively for 28 days. Treatment of diabetic rats with *S. persica* twig powder in G3 and G4 greatly recovered all biochemical and histological damage and returned all nearly to the normal.

Another study by (Haddad El Rabey et al., 2018) concluded that *S. persica* leaf aqueous extract has hypoglycaemic, antioxidant and hypolipidemic activities in alloxan-induced diabetic male rats. All biochemical parameters and the injured liver, kidney and pancreas tissues were recovered nearly to the normal as in the negative control group.

An evaluation by (Saeedi Borujeni et al., 2019) to the actions of various fractions of *S. persica* extract on, lipid peroxidation, and insulin sensitivity of diabetic rats has been made. The test groups took an oral dose of 200, 400, and 600 mg/kg of crude, aqueous, and ethyl acetate fractions of *S. persica* extract. After twenty-one days, the fasting blood glucose, lipid profiles, lipid peroxidation, insulin sensitivity, liver enzymes levels, liver histopathology, and body weight alteration were recorded. Their results gave a proof that the *S. persica* extract, especially aqueous one, can help in diabetes and can be recommended as a complementary therapy for disease management.

Based on the above studies, *S. persica* displays dose-dependent hypoglycemic and hypolipidemic activities. The mechanism of enhancing the lipid profile has not been clarified yet. The proposed mechanisms include increasing cholesterol degradation, prevent cholesterol absorption in the intestine, an increase in cholesterol removal as bile acids or other sterols, interference with lipoproteins (Galati et al., 1999), and reducing LDL cholesterol levels by flavonoids, which significantly increase the LDL receptor mRNA levels, leading to increasing the hepatic uptake and degradation of LDL cholesterol. Therefore, deeper work is requested to elucidate the hypolipidemic mechanisms of *S. persica*.

### 3.14 Cytotoxicity and antitumor activities

An investigation of the cytotoxic potential of *S. persica*, using an agar overlay method, on the gingiva and other periodontal parts didn’t reveal cytotoxic effect by a freshly cut and freshly used Arak. However, after 24 h of using, the same plant was shown to contain bad ingredients. From these results, it is better that the used part of the miswak is cut after 1 day of use and a fresh part is prepared. The cytotoxicity became obvious in this research just after 24 h due to depending of the agar overlay method on the diffusion of the medical agent into agar. In addition, the method does not provide a direct contact between cells and the test solution (Al-Samh and Al-Nazhan, 1997).

Currently, there is great interest in discovering new anticancer agents from natural products. Fortunately, *S. persica* can be helpful in this area. Al Bratty *et al*. have recently evaluated the cytotoxic properties of the fruits ethanol extract by MTT assay using the breast MCF7, ovary A2780, and colon HT29 cells (Al Bratty et al., 2020). They found that the fruit extract was selective against the ovarian and colon cancer cells compared to normal fibroblast cells [MRC5] as it showed IC_50_ values 17.50, 8.35, and 5.12 μg/mL versus MCF7, A2780, and HT29 cells, respectively.

Different extracts of *S. persica* sticks and the bark were screened and the petroleum ether fraction was the most active with IC_50_ values of 43.6 μg/mL against HepG2 cell line, 44.3 μg/mL against MCF7 cell line, 19.87 μg/mL against A549 [the lung carcinoma] cell line, and 10.2 μg/mL against HCT116 [the colon carcinoma] cell line, which were significantly lower than the cytotoxic concentration for normal green African monkey kidney cells [Vero] [IC_50_ = 379 μg/mL], as has been observed by (Ibrahim et al., 2011a).

Moreover, (Hammad et al., 2014) have documented that *S. persica* aqueous extract, examined at 11.25, 13.50, and 15.75 mg/mL, displayed significant cytotoxic effects on both PE/CA-PJ15 [oral squamous cell carcinoma] and DOK [oral epithelial dysplasia] cell lines at a concentration lower than the significant cytotoxic concentration for the normal periodontal ligament fibroblast cell line.

Therefore, these previous studies suggest the preventive and therapeutic anticancer action of *S. persica*. Also, stigmast-5,22-dien-3β-ol was purified by (Iyer and Patil, 2012) from the plant and shown to contribute to its antitumor activity. The antioxidant properties of the plant may also help in its antitumor activity. However, protection mechanisms against free radicals incorporated in cancer etiology still require clarification. It is important to mention that the potential activity of the plant against other cancer cell lines has not been studied.

Moreover, future studies are required to validate its potential in oral cancer prevention and/or treatment, considering the common use of *S. persica* in oral care. Another potential area of research is the testing of potentiating effects of *S. persica* extracts or their major compounds on chemotherapeutic agents.

### 3.15 Possible activity against COVID-19

In Dec. 2019, an outbreak of coronavirus disease [COVID-19] started from Wuhan, China and spread around 210 countries within weeks. It was named coronavirus 2 [SARS-CoV-2] and induced serious respiratory syndrome that could cause mortality. Among the hopeful therapeutic strategies against the viral infection is using molecular docking and search for natural enzyme inhibitors to produce compounds with slight adverse effects. COVID-19 virus main protease has important role in facilitating viral transcription and replication, so it may be a possible target for antiviral agents. Metabolic profiling of the aerial parts aqueous extract of *S. persica* documented eleven known flavonol glycosides using LC-HRESIMS. All of them revealed significant binding stability at the N3 binding site to different degrees, except isorhamnetin-3-*O*-β-D-glucopyranoside, when compared with the currently used COVID-19 main protease inhibitor, darunavir.

The results show that the basic flavonol nucleus has the activity itself based on structural similarity between the entitled flavonoids. Moreover, the presence of a rutinose moiety at the 3 position of ring C and absence of an O-Me group in ring B of the flavonol structure could enhance the binding stability (Owis et al., 2020). This research gives a scientific proof that regular use of meswak can help in protection against COVID-19 as it releases active flavonoids in the saliva.

## 4 Analytical standardization methods

To address the increasing demand for developing pharmaceutical products with potent biological activities, many analytical methods have been developed to standardize the extracts. Benzyl cyanide, 2-Methoxy-4-(prop-2-en-1-yl) phenol, 2-isopropyl-5-methylphenol, 4-methyl-2-propan-2-ylphenol, 1,3,3-Trimethyl-2-oxabicyclo [2.2.2] octane, isoterpinolene, and β-caryophyllene were identified by (Alali and Al-Lafi, 2003) using gas chromatography (GC)–mass spectrometry examination of the *S. persica* leaves’ volatile oil. The aroma toxicity was assessed using a brine shrimp lethality test, which resulted in a 50% lethal concentration (LC_50_) > 1,000 ppm. Using a disc diffusion test, comparable antibacterial effects on several different oral aerobic bacteria were observed for a leaf extract, when compared with known antibiotics.

Interestingly, a high-performance thin-layer chromatography method has recently been reported for measurement of a major antimicrobial compound, benzyl isothiocyanate, in extracts of *S. persica* roots and in dental care herbal products. A modified HPTLC method, developed by (Abdel-Kader et al., 2017a, Abdel-Kader et al., 2017b, Abd El Kader et al., 2017c) using glass-coated silica gel 60 F_254_ plates and a mobile phase [*n*-hexane–ethyl acetate 9:1], provided compact spots at *R*_F_ of 0.61 ± 0.01. The developed plate was scanned and quantified densitometrically at 191 nm. A significant linear relationship between the peak area and the amount of benzyl isothiocyanate was observed in the range of 100–600 ng per band [*r*^2^ = 0.9991] using linear regression analysis. Owing to ICH [the International Conference on Harmonization] guidelines, the method was valid, based on its accuracy, precision and robustness. This procedure will be helpful to validate the efficacy of miswak extracts and of dental care herbal formulations claimed to contain it.

Another method for benzyl isothiocyanate quantitative analysis was developed by (Abdel-Kader et al., 2017a, Abdel-Kader et al., 2017b, Abd El Kader et al., 2017c) using a reversed-phase C_18_ high-performance liquid chromatography column. This technique can be used for measuring amount of benzyl isothiocyanate in miswak extracts, a dental care powder, mouth wash, and toothpaste, claimed to contain it. Separation was arranged by a reversed-phase C_18_ [250 × 4.6 mm, 5 μm] column with a mobile phase comprising acetonitrile and water [1:1]. The detection was done at 190 nm using an ultraviolet–visible detector. The flow rate was constant at 1 mL/min. According to the ICH guidelines, this assay was validated. The proposed procedure was reported to be selective, easy, specific, and reliable and thus can be used routinely in quality control analysis of various commercial herbal care products.

Moreover, using GC, (Abdel-Kader et al., 2018) have developed an accurate, simple, precise, and sensitive procedure for quantitative analysis of benzyl isothiocyanate in plant extracts and dental care herbal products claimed to contain miswak. Helium as a carrier gas at a flow rate of 0.74 mL/min and an Rtx column [30.0 m × 0.25 mm ID, 25 mm thickness] were used. Under the described conditions, the retention time of the benzyl isothiocyanate standard was recorded at 13.470 min. A good linear relationship between the peak height and concentration of benzyl isothiocyanate was shown in the range of 10–50 mg/mL [*R*^2^ = 0.9971] by linear regression analysis. The validation criteria set by the ICH guidelines for the accuracy, precision and robustness were met by this method.

These GC methods of analysis have been suggested to be suitable and valuable for benzyl isothiocyanate determination in siwak extracts and other products containing siwak extracts. The efficacy of the products may be evaluated based on the amount of benzyl-isothiocyanate.

Recently, (Abdel-Kader et al., 2019b) have investigated the effect of extraction conditions on the amount of benzyl isothiocyanate which is the major antimicrobial component in meswak. They applied both cold and hot extraction with different solvents and quantified concentration of benzyl isothiocyanate by HPLC and HPTLC. They noticed that cold extraction of the fresh samples with chloroform offers the maximum amount of benzyl isothiocyanate. They concluded that drying process leads to great loss of the major active antimicrobial component of miswak.

Moreover, (Abdel-Kader et al., 2019a) have examined hydroxylated solvents’ effect on the active components of *S. persica* root. They found that only solvents with hydroxyl groups reacted with benzyl isothiocyanate and gave products similar to those isolated from the alcohol extracts. They concluded that *S. persica* extraction with hydroxylated solvents will modify the structure of the active compound benzyl isothiocyanate causing loss of antimicrobial activity.

## 5 Industrial applications of miswak

### 5.1 Industrial oil production

*S. persica* can be an important seed oil crop for alkali and saline soils because the seeds contain 40–45% of oil rich in industrially important lauric [C_12_] and myristic [C_14_] acids. Reddy and his group have attempted to evaluate behavior of this plant on saline and alkali soils and reported that the species could grow on both soil types (Reddy et al., 2008). However, a significantly higher spread, height, and seed yield were observed for plants growing on saline soil than for those growing on alkali soil, with no significant changes in the oil constitution. They have suggested *S. persica* as a crop for industrial oil production with economic and ecological benefits on both saline and alkaline soils.

### 5.2 Bio-preservation of food

Recently, natural antimicrobial compounds have been used to prevent microbial contamination without changing the good properties of food goods (Woraprayote et al., 2016) . Efficacy of *S. persica* root aqueous extracts (roots soaked for 24 and 48 h in water) as a natural food preservative for chicken burgers has been observed by (Abou-Zaid et al., 2015) who evaluated effects of the extracts on the microbial profile, chemical composition, and organoleptic properties. The data showed a concentration-dependent activity, with a greater reduction in the total viable microbial count in chicken burgers reported for the 48-h extract than for the 24-h extract. In addition, after increasing the concentration of the extract from 12.5% to 50%, coliform bacteria growth rate in chicken burgers was reduced.

In a recent research by (Saddiq and Alkinani, 2019), the fungicidal effect of *S. persica* aquatic root extract has been evaluated in terms of inhibition zone and radial growth rate against three pathogenic *Aspergillus* species, namely, *A. niger*, *A. flavus,* and *A. fumigatus in vitro*. The outcomes showed that the plant extract [50 and 100 mg/mL] suppressed the tested fungal species growth. The higher concentration [100 mg/mL] of the tested extract prohibited the growth rate of the entitled *Aspergillus* species after six days’ exposure period. This may suggest use of this extract as a possible antifungal agent vs. aspergillosis.

Therefore, *S. persica* can be suggested as an economical and safe natural food preservative. More studies are recommended to investigate its use in other food products such as fruits, vegetables and meat products.

### 5.3 Functional food development

In recent decades, food impact on health has increased. Besides satisfying the hunger, appetite sensations, and basic nutritional requirements, consumer interest has shifted toward a greater role of food in preventing the risk of diseases and improving the quality of life. Therefore, a diversity of new functional food products has continuously been developed to gain consumers' acceptance, expectations, and requests (Siro et al., 2008).

Since the biological potential of *S. persica* has been proven by many reports, new functional foodstuffs can be manufactured by including various parts of *S. persica* into functional dairy and fruity yogurts, beverages, and cereals. For centuries, many parts of *S. persica* have been used orally for curing several disorders as mentioned before.

Thus, to treat scurvy, leaf juice can be taken (Upadhyay et al., 2007). Asthma and malaria can be treated by a decoction of leaves (Shah et al., 2017). For curing urinary retention and bilharzia, an infusion of leaves is administered orally. In addition, female infertility can be improved by taking a decoction of roots (Chhabra et al., 1991). Taking the traditional oral use of many parts of *S. persica* into consideration with their documented biological activities, we recommend that *S. persica* is included in functional foodstuffs with help of other delicious ingredients, to make it acceptable by customers.

### 5.4 Nanotechnology

Nanotechnology can be considered a research field of the modern era. Interestingly, green synthesis is the most demanded and reasonable method, although nanoparticles can also be developed by chemical and physical methods (Neelima et al., 2016). The plant extract-mediated development of NPs may simplify the large-scale biosynthesis of metallic NPs since it is easy to use and cheap and does not use hazardous chemicals, solvents, and advanced laboratory facilities (Khan et al., 2017). Silver nanoparticles (Ag-NPs) are the most important metallic NPs because of their various applications in different fields.

Both chemical and green synthesis of Ag-NPs, with an aqueous solution of an *S. persica* root extract as a bio-reductant, were evaluated by (Shaik et al., 2016). The extract alone (10–300 μg/mL) didn’t have any antibacterial activity. Since the concentration used for the development was too small to exert its own antibacterial activity, the role of the extract was mainly to serve as a reductant for the process. However, the extract displayed significant effects on the antibacterial action of the green-synthesized Ag-NPs. The influence of the volume of the plant extract on the size of NPs and their dispersion qualities has been demonstrated in this study.

A study by (Barzegar et al., 2017) have succeeded in synthesis Ag-NPs with ethanol leaf extract of *S. persica.* A stronger antifungal activity versus *Aspergillus niger* and *Penicillium digitatum* was displayed by these green-synthesized NPs than that displayed by the ethanol extract of *S. persica* leaves. In addition, Ag-NPs synthesized using the plant twig extracts were examined against *P. gingivalis* (Neelima et al., 2016). A greater antibacterial activity was shown by Ag-NPs produced with freshly-prepared extracts than by those produced with 1-month-old extracts.

Recently, (Miri et al., 2020) have documented the biosynthesis of cerium oxide nanoparticles (CeO_2_ -NPs) using *s. persica* aqueous extract. These nanoparticles play an important role in enhancing technologies such as polishing, harmful industrial dyes degradation and even curing some diseases.

Also, (Verma et al., 2020) have studied fabrication of zinc oxide NPs using *S. persica* root extract as a reducing agent and observed that changing the extraction solvent changes ZnO NPs morphology. These ZnO NPs with varying morphology can be used as medical agents, biosensors, photochemistry and electrical applications.

Based on the above data, we can consider potential applications of metallic NPs synthesized with *S. persica* in different areas. We recommend future studies to explore the total possible biological actions of these green-synthesized NPs and to incorporate the bioactive components of *S. persica* into NPs.

### 5.5 Miscellaneous industrial applications

The ability of *S. persica* root powder as a green bio sorbent for barium and strontium purification from wastewater and radioactive wastes have recently been examined by (Hassan et al., 2020). In addition, (Ali et al., 2018) have recently succeeded in converting vegetable seed oil of *S. persica* into biodiesel that satisfy the international biodiesel standard [ASTM D6751].

Here in table 1, we are summarizing the most important biological action of each extract/ fraction or pure compound from *Salvadora persica* reported until now to the best of our knowledge.

**Table 1:** Summary of the most important biological actions of the plant.

**Part of the plant**	**The active extract, fraction or pure compund**	**The biological effect or action**	**Mechanism of action or target**	**Reference**
Roots	Ethanol extract and its purified persicaline compound.	Antioxidant.	Not identified.	(Farag et al., 2018).
Roots.	Benzyl isothiocyante and its hydroxyl and methoxy derivatives	Antimicrobial	Suppress the bacterial redox system by damaging the membrane potential.	(Abd El Kader et al., 2017c).
Fruits	Fruits extract	Antimicrobial activity for *Streptococcus* mutans.	Inhibits cariogenic bacteria growth and acid production.	(Al Bratty et al., 2020).
Roots	Essential oils	Antimicrobial comparable to that of chlorhexidine digluconate.	Inhibits cariogenic bacteria growth and acid production.	(Khan et al., 2020).
Not specified.	Apigenin, luteolin, astragalin and kaempferol-3-*O*-rhamnoside from alcoholic extract.	Anti-MRSA [Methicillin-resistant Staphylococcus aureus].	Not identified	(Ghoneim et al., 2019).
Twigs	Methanol extract.	Wound healing effect.	By antioxidant and antimicrobial properties.	(Tatke et al., 2017)
Roots	Aqueous extract.	Antiprotozoal activity.	Not identified.	(El-Bali et al., 2020).
Leaves	Ethyl acetate extract.	Anti-inflammatory.	May be due to high concentration of flavonoids.	(Rajesh et al., 2010).
Sticks	crude ethanol extract and its ethyl acetate fraction.	Anti-inflammatory.	May be due to high concentration of flavonoids.	(Ibrahim et al., 2011b).
Roots	Crude hydro-alcholic extract	Analgesic activity.	Not identified.	(Hooda and Pal, 2017).
Twigs	a lyophilized decoction	Antiulcer	Not identified.	(Sanogo et al., 1999).
Stems	a lyophilized decoction	Antiulcer	Not identified.	(Monforte et al., 2001).
Stems	Total extract	Antidepressant	Not identified.	(Monforte et al., 2002).
Roots	Aqueous extract.	Hypoglycemic and hypolipidemic.	Not identified.	(Khan et al., 2014).
Twigs	Powder.	Hypoglycemic and hypolipidemic.	Not identified.	(El Rabey et al., 2017).
Leaves	Aqueous extract.	Hypoglycemic and hypolipidemic.	Not identified.	(Haddad El Rabey et al., 2018).
Fruits	Ethanol extract.	Anticancer.	Cytotoxic effect.	(Al Bratty et al., 2020).
Bark	Petroleum ether extract	Anticancer	Cytotoxic effect.	(Ibrahim et al., 2011a).
The aerial parts	Aqueous extract	May help against COVID-19	COVID-19 main protease inhibitor.	(Owis et al., 2020).

## 6 Conclusion

From the above data it is logic to report that each part of *S. persica* should be suspected to contain pharmaceutically important phytochemicals that can contribute to the health of humans and animals. A future investigation may result in the discovery of new bioactive compounds in *S. persica*, leading to the formulation of more potent drugs and/or new bioproducts especially if the method of extraction or purification differs from the common methods as reported with purification of persicaline by acid-base extraction method not only the alkaloid-rich fraction.

It is clear that *S. persica is* still giving researchers great results in many fields especially natural products chemistry and their biological potentials not only for oral care. Also, we strongly recommend further studies to estimate the impact of extraction solvents on the quality and quantity of the active compounds in addition to mechanism of actions of the extracts and purified compounds or fractions.

For instance, the future possible area of research is to isolate the active antiviral compounds which could assist in discovering drugs to target the current SARS-CoV-2 pandemic.

Moreover, deeper studies are highly recommended to discover the potential antiprotozoal and antiosteoporosis effects of different parts of *S. persica*, and more trials should be conducted to isolate bioactive compounds responsible for these effects.

Thus, it is highly recommended to extend research on this amazing plant for more benefits for humanity.

## Declaration of Competing Interest

The authors declare that they have no known competing financial interests or personal relationships that could have appeared to influence the work reported in this paper.
